# Telomere length as biomarker of nutritional therapy for prevention of type 2 diabetes mellitus development in patients with coronary heart disease: CORDIOPREV randomised controlled trial

**DOI:** 10.1186/s12933-024-02175-5

**Published:** 2024-03-16

**Authors:** Ana Ojeda-Rodriguez, Oriol A. Rangel-Zuñiga, Antonio P. Arenas-de Larriva, Francisco M. Gutierrez-Mariscal, Jose D. Torres-Peña, Juan L. Romero-Cabrera, Alicia Podadera-Herreros, Helena García-Fernandez, Esther Porras-Pérez, Raul M. Luque, Stefanos N. Kales, Pablo Perez-Martinez, Javier Delgado-Lista, Elena M. Yubero-Serrano, Jose Lopez-Miranda

**Affiliations:** 1 Lipids and Atherosclerosis Unit, Internal Medicine Unit, Reina Sofia University Hospital, Cordoba, 14004 Spain; 2 Department of Medical and Surgical Science, University of Cordoba, Cordoba, 14004 Spain; 3 Maimonides Biomedical Research Institute of Cordoba (IMIBIC), Av. Menendez Pidal, s/n, Cordoba, 14004 Spain; 4 CIBER Fisiopatologia de la Obesidad y Nutricion (CIBEROBN), Instituto de Salud Carlos III, Madrid, 28029 Spain; 5 Department of Cell Biology, Physiology and Immunology, University of Cordoba, Cordoba, 14004 Spain; 6 Department of Environmental Health, Harvard T.H. Chan School of Public Health, Boston, MA USA; 7 Department of Occupational Medicine, Cambridge Health Alliance, Harvard Medical School, Cambridge, MA USA

**Keywords:** Cardiovascular disease, Telomere attrition, Aging, Lifestyle intervention

## Abstract

**Background:**

Telomere Length (TL), a marker of cellular aging, holds promise as a biomarker to elucidate the molecular mechanism of diabetes. This study aimed to investigate whether shorter telomeres are associated with a higher risk of type 2 diabetes mellitus (T2DM) incidence in patients with coronary heart disease; and to determine whether the most suitable dietary patterns, particularly a Mediterranean diet or a low-fat diet, can mitigate the development of diabetes in these patients after a follow-up period of five years.

**Methods:**

The CORonary Diet Intervention with Olive oil and cardiovascular PREVention study (CORDIOPREV study) was a single-centre, randomised clinical trial done at the Reina Sofia University Hospital in Córdoba, Spain. Patients with established coronary heart disease (aged 20–75 years) were randomly assigned in a 1:1 ratio by the Andalusian School of Public Health to receive two healthy diets. Clinical investigators were masked to treatment assignment; participants were not. Quantitative-PCR was used to assess TL measurements.

**Findings:**

1002 patients (59.5 ± 8.7 years and 82.5% men) were enrolled into Mediterranean diet (*n* = 502) or a low-fat diet (*n* = 500) groups. In this analysis, we included all 462 patients who did not have T2DM at baseline. Among them, 107 patients developed T2DM after a median of 60 months. Cox regression analyses showed that patients at risk of short telomeres (TL < percentile 20th) are more likely to experience T2DM than those at no risk of short telomeres (HR 1.65, *p-value* 0.023). In terms of diet, patients at high risk of short telomeres had a higher risk of T2DM incidence after consuming a low-fat diet compared to patients at no risk of short telomeres (HR 2.43, 95CI% 1.26 to 4.69, *p-value* 0.008), while no differences were observed in the Mediterranean diet group.

**Conclusion:**

Patients with shorter TL presented a higher risk of developing T2DM. This association could be mitigated with a specific dietary pattern, in our case a Mediterranean diet, to prevent T2DM in patients with coronary heart disease.

**Trial Registration:**

Clinicaltrials.gov number NCT00924937.

**Supplementary Information:**

The online version contains supplementary material available at 10.1186/s12933-024-02175-5.

## Introduction

The growing prevalence of type 2 diabetes mellitus (T2DM) as well as cardiovascular disease (CVD) represents a significant global public health challenge. Notably, coronary heart disease (CHD) is as a major cause of death and disability worldwide [1]. The relationship between T2DM and CHD is well-established, and patients with both diseases may have poorer prognosis, experiencing increased complications and a higher risk of adverse cardiovascular events. The pathophysiological mechanisms underlying T2DM, characterized by insulin resistance and impaired beta-cell function, exert detrimental effects on CHD outcomes, contributing to the progression of atherosclerosis, impaired blood flow regulation and increased inflammatory status [2].

Prevention through lifestyle is the cornerstone of reducing the risk of developing T2DM, especially by increasing physical activity levels and improving diet quality [3]. In this context, determining promising biomarkers to identify individuals at high risk of developing T2DM as well as to assess the efficacy of targeted interventions for prevention is of paramount importance.

Over the last decade, telomere length (TL) has emerged as a potential biomarker to understand the molecular processes that underlie diabetes [4]. Telomeres are repetitive nucleotide sequences (5’ – TTAGGG – 3’) at the end of chromosomes, which provide stability and protection of genetic material. In each cell division, a small telomeric DNA fragment is lost due to the inability of telomerase activity in most cells. In addition, the high guanine content of telomeres makes them susceptible to reactive oxygen species, commonly generating 8-oxoguanine [5]. Consequently, TL naturally reaches a critical size, which can be expedited by a variety of intrinsic and environmental factors through the upregulation of oxidative stress levels [6]. In this sense, TL is often considered a biomarker for cellular aging, and has been associated with increased risk of several diseases, such as CVD [7], obesity [8], diabetes [9–11], and cancer [12]. Emerging studies have suggested that cellular aging reflected by TL could be related to the development of T2DM, marked by elevated levels of oxidative stress induced by hyperglycaemia [4]. Despite these findings, the absence of interventional studies assessing how specific dietary approaches could influence the association between TL and the risk of T2DM incidence, especially in the context of secondary cardiovascular prevention, means that this possible association remains unclear.

Based on this, our study aims to assess whether shorter telomeres is associated with a greater risk of T2DM incidence in patients with CHD. Additionally, we aim to determine whether the most suitable dietary patterns, particularly a Mediterranean diet rich in extra virgin olive oil, or a low-fat diet rich in complex carbohydrates, can mitigate the development of diabetes in these patients after a follow-up period of five years.

## Materials and methods

### Study population

The present study was carried out as part of the CORDIOPREV study, which stands for the CORonary Diet Intervention with Olive oil and cardiovascular PREVention study (Clinicaltrials.gov number NCT00924937). This research was conducted at Reina Sofia University Hospital in Córdoba, Spain. The study design involved a single centre, randomized, single-blind, and controlled dietary intervention. A total of 1002 patients with CHD were included, and detailed information regarding the rationale, study methods, inclusion and exclusion criteria, cardiovascular risk factors, and baseline characteristics of the patients have been published recently [13]. In brief, eligible patients included men and women between the ages of 20 and 75 years, who had established CHD, were free of clinical coronary events in the previous six months, were able to follow a long-term dietary intervention, and had no severe illnesses or an expected life expectancy lower than the length of the study. The upper age limit was set based on the life expectancy at the conception of the trial (2007) and it was in line with the usual practice in contemporary long-term cardiovascular studies.

Informed written consent was obtained from all participants prior to their inclusion in the study. The study protocol received approval from the Human Investigation Review Committee at Reina Sofia University Hospital, following the institutional and Good Clinical Practice guidelines.

The primary objective of the CORDIOPREV study is to evaluate the efficacy of a Mediterranean diet as compared with a low-fat diet to prevent clinical events and mortality in patients with previous CHD through a long-term follow-up study. Here, we report the results of one secondary outcome of the CORDIOPREV study: T2DM incidence. In this work, we included 462 out of 1002 patients who had not been diagnosed with T2DM at the study’s outset. Of these Non-T2DM patients at baseline, 107 patients developed T2DM (Incident-T2DM) according to the American Diabetes Association (ADA) diagnostic criteria [14], after a median follow-up of 60 months. In this study, 19 patients were excluded because we did not have their TL data; thus, the final sample consisted of 338 Non-T2DM and 105 Incident-T2DM (Additional Fig. 1). Every year, T2DM incidence was assessed according to the ADA T2DM criteria: fasting plasma glucose ≥ 126 mg/dL or 2 h plasma glucose in the 75 g oral glucose tolerance test ≥ 200 mg/dL or glycosylated haemoglobin (HbA1c) levels ≥ 6.5%. In the absence of unequivocal hyperglycaemia, diagnosis requires two abnormal test results from the same sample or in two separate test samples [14].

The sample size and the power calculation of this sub-study were calculated based on of the following assumptions: a T2DM incidence rate in the Mediterranean diet group of 10%, and 17% in the low-fat group and a statistical power of 80%, with α of two tails = 0.05. With these assumptions and assuming a 10% of estimated loss, the necessary sample size was established from 163 patients in each of the dietary intervention groups. In the present work, all the subjects who had not been clinically diagnosed with T2DM at baseline were included (*n* = 462; 216 Low-fat Diet and 246 Mediterranean Diet). Of this group, all 107 subjects who development T2DM were included (42 Low-fat Diet and 65 Mediterranean Diet). The remaining 355 subjects were not diagnosed of T2DM (174 Low-fat Diet and 181 Mediterranean Diet) (Additional Fig. 1).

### Randomisation and masking

Randomisation was performed by the Andalusian School of Public Health (1:1 ratio). The dietitians were the only members of the intervention team to know about each participant’s dietary group. Briefly, the randomisation was based on the following variables: sex (male, female), age (< 60 and ≥ 60 years old) and previous myocardial infarction (yes, no). Details about randomisation have been previously reported and summarized [15].

### Dietary intervention

Patients were randomized to receive one of two different healthy dietary models: (1) a Mediterranean diet, with a minimum 35% of calories from fat (22% monounsaturated, 6% polyunsaturated and < 10% saturated fatty acids), 15% proteins and a maximum of 50% carbohydrates, and (2) a low-fat diet recommended by the National Cholesterol Education Program, with < 30% of total fat (12–14% monounsaturated, 6–8% polyunsaturated and < 10% saturated fatty acids), 15% proteins and a minimum of 55% carbohydrates. In both diets, the cholesterol content was adjusted to < 300 mg/day. The present study was conducted over a follow-up period of five years. Details about diets have been previously reported and summarized [15].

Patients underwent individual face-to-face interviews with a nutritionist at baseline and annually to complete a 137-item semiquantitative food frequency questionnaire, previously validated in Spain [16]. Each year, the 14-item MEditerranean Diet Adherence Screener [17] was used to measure adherence to the Mediterranean diet and a 9-item dietary screener was used to evaluate adherence to the low-fat diet. In addition, both intervention groups received identical intensive dietary counselling. Nutritionist-conducted personalized one-on-on interviews at baseline and every six months. Furthermore, quarterly educational collectively sessions were held with up 20 participants per session and separate sessions for each group.

### Laboratory measurements

After a 12-h overnight fast at the beginning of the study and once a year during the follow-up period, venous blood samples were collected from the participants in EDTA tubes (final concentration of 0.1% EDTA) and plasma was separated from the red cells by centrifugation at 1500 × g for 15 min at 4 °C and immediately frozen at − 80 °C. The biochemical measurements were performed at the Reina Sofia University Hospital by personal who were blinded to the interventions. Lipid variables were assessed with a DDPPII Hitachi modular analyser (Roche, Basel, Switzerland) using specific reagents (Boehringer-Mannheim, Mannheim, Germany). Plasma triglycerides and cholesterol concentrations were assayed by enzymatic procedures. High-density lipoprotein-cholesterol (HDL-c) were measured following precipitation of a plasma aliquot with dextran sulfate-Mg2+. Low-density lipoprotein-cholesterol (LDL-c) concentration was calculated by the Friedewald equation, using the following formula: LDL-c = Total Cholesterol − (HDL-c + Triglycerides/ 5). Glucose measurements were performed by the hexokinase method. High sensitive C-Reactive Protein (hs-CRP) was determined by ELISA techniques (BioCheck, Inc., Foster City, CA, USA).

### DNA isolation from blood samples

DNA was isolated from buffy coat fraction through the salting-out method [18], using 10 mL of Montreal-Baltimore buffer (0.32 M sucrose, 0.1 mM Tris-HCl, pH 7.5, 0.025 mM MgCl2, 1% Triton X-100) and mixing and centrifuging to separate the nuclear fraction. The nucleic pellet was homogenized with 3 mL of nuclei lysis buffer (10 mM Tris-HCl, pH 8.2, 2 mM EDTA, 0.4 M NaCl) and 10% SDS and proteinase K. The DNA was precipitated with 6 M NaCl and washed with 100% ethanol. Finally, the genomic DNA was extracted and resuspended in 500 µL of 1× TE buffer. DNA purity and concentration were evaluated by spectrophotometry using NanoDrop ND-2000 (ThermoFisher, Waltham, MA).

### Quantitative PCR analysis of TL

TL was determined using the Cawthon method by qPCR [19]. For all samples, we estimated the relative ratio of telomere repeat copy number (T) normalized against a single copy gene, the homo sapiens ribosomal protein L13a gene *RPL13a* (S). Results for each PCR were relativized to a standard curve, built using a reference DNA sample. The standard curves for telomere and *RPL13a* gene PCR consisted of eight DNA reference standards (1–25ng). All PCRs were performed with the use of iQ5-BIORAD thermal cycler and SensiFAST SYBR Lo-ROX kit (Bioline). The coefficient of variation (%CV) was 9.32% for the telomere repeat copy number and 6.76% for the single copy gene copy number. The thermal cycler profile for both amplicons began with 95 °C incubation for 3 min to activate the polymerase, followed by 40 cycles of 95 °C for 5 s, 54 °C for 15 s. The reaction mix composition was identical except for the oligonucleotide primers: 20-ng template DNA, 1× SensiFAST SYBR Lo-ROX, 200-nM reverse primer, and 200-nM forward primer. The final volume for each PCR reaction was 20 µL. The primer sequences were (5′→ 3′):

TeloFw, CGGTTTGTTTGGGTTTGGGTTT GGGTTTGGGTTTGGGTT; TeloRw, GGCTTGCCTTACCC TTACCCTTACCCTTACCCTTACCCT; RPL13aFw, CCTGGAGGAGAAGAGGAAAGAGA; RPL13aRw, TTGAGGACCTCTGTGTATTTGTCAA.

DNA extraction and TL measurements were performed at baseline and at the 4-year time point. While assessing TL at additional time points could provide further valuable insights, we did not observe statistically significant changes in TL at 4-year time point within our study. In this work, we evaluate the role of TL at baseline as a marker for identifying individuals at risk of short TL (TL < percentile 20th).

### Statistical analyses

The statistical analyses were performed with STATA 14 (STATA Corp., TX, USA). We used the mean and standard error of the mean (mean ± SEM) for continuous variables, and percentages for categorical variables. All p-values were 2-tailed, and a *p* < 0.05 was considered statistically significant.

The definition of the shortest TL, rather than the average TL, is associated with telomere dysfunction and cellular survival limitation [20, 21]. In this way, patients were classified according to the risk of short TL at baseline, defined as a TL below the 20th percentile and no risk as a TL above the 20th percentile, as it was previously reported [22–24].

Student’s unpaired was used for comparison between groups. Categorical variables were compared using Chi-Square tests. We used Fisher’s exact test to evaluate the relationship between participant’s T2DM incidence and risk of short TL. Mosaic plots have been applied to show the relationship between T2DM incidence and the risk of short TL, in which height of bars represents proportions of participants with high or low risk of short telomere, and the width of each bar is proportional to T2DM incidence. Logistic regression model was run to assess the risk of T2DM incidence according to the risk of short telomere. We used the Cox proportional hazards regression to test the potential predictive value of TL and the potential role of TL to select dietary model to decrease the risk of developing diabetes. In addition, we conducted statistical analysis to assess the interaction by diet group and risk of short TL in the Cox regression model. The regression analyses were adjusted by age, gender, body mass index (BMI), waist circumference (WC), HbA1c, HDL-c,triglycerides and family history of both diabetes and early coronary heart disease. As additional analyses, receiver operating characteristic curve (ROC) analysis was run for assessing the potential for TL to classify the population in Incident-T2DM and Non-T2DM patients. We performed two models: the first based on clinical variables (BMI, age, gender, triglycerides and statins use), the second model with added TL.

## Results

### Baseline characteristics of the participants

Baseline body weight, BMI, WC, HbA1c, fasting glucose, and insulin levels were higher in the Incident-T2DM patients than in Non-T2DM patients (all *p* < 0.05) (Additional Table 1). Baseline characteristics of the patients classified according to the risk of short telomeres (< 20th percentile) are showed in Table 1. Similar differences were found between Incident-T2DM and Non-T2DM patients with no risk of short telomeres with higher baseline BMI, WC, HbA1, glucose, and insulin levels in Incident-T2DM patients compared to Non-T2DM patients (all *p* < 0.05). However, in patients at risk of short telomeres, Incident-T2DM patients showed lower HDL-c levels, and higher HbA1 and fasting glucose levels than Non-T2DM patients (all *p* < 0.05) (Table 1).

**Table 1:** Baseline characteristics of the participants according to the risk of short telomeres

	Non-Risk of Short telomeres (n = 356)			Risk of Short telomeres (n = 87)		
	**Non-T2DM** (n = 279)	**Incident-T2DM** (n = 77)	***p-value***	**Non-T2DM** (n = 59)	**Incident-T2DM** (n = 28)	***p-value***
Men/Women (n)	235/44	62/15	0.288	53/6	24/4	0.574
LowFat/MeDiet (%)	50/50	38/62	0.054	39/61	46/54	0.510
Age (years)	57.25 ± 0.55	59.20 ± 1.07	0.103	57.66 ± 1.37	58.42 ± 1.47	0.731
Weight (kg)	82.21 ± 0.76	85.33 ± 1.84	0.075	82.82 ± 1.90	86.63 ± 2.36	0.239
BMI (kg/m2)	29.84 ± 0.24	31.45 ± 0.58	**0.004**	29.59 ± 0.57	31.38 ± 0.76	0.070
WC (cm)	101.12 ± 0.62	105 ± 1.36	**0.005**	103.63 ± 1.47	106.22 ± 1.77	0.295
HDL-c (mmol/L)	44.19 ± 0.59	45.36 ± 1.31	0.384	46.29 ± 1.27	38.41 ± 1.21	**< 0.001**
LDL-c (mmol/L)	91.12 ± 1.49	96.48 ± 3.42	0.115	91.24 ± 3.39	86.22 ± 3.65	0.3734
hsCRP (nmol/L)	2.38 ± 0.21	2.79 ± 0.32	0.335	3.04 ± 0.51	3.08 ± 0.67	0.954
Triglycerides (mmol/L)	118.56 ± 3.61	133.51 ± 8.02	0.066	122.54 ± 7.98	124.93 ± 10.87	0.863
HbA1c (%)	5.85 ± 0.02	6.02 ± 0.04	**< 0.001**	5.85 ± 0.04	6.05 ± 0.07	**0.014**
Glucose (mmol/L)	92.41 ± 0.60	95.46 ± 1.22	**0.021**	92.92 ± 1.21	98.0 ± 2.02	**0.025**
Insulin (nmol/L)	8.25 ± 0.35	10.43 ± 0.82	**0.007**	8.85 ± 0.70	11.24 ± 1.00	0.055

Continuous variables are represented as means ± SE. Categorical variables are expressed as percentage. Variables were compared using the analysis t-test. Bold *p-values* denote statistical significance (*p-value*<0.050)

*Abbreviations* BMI Body Mass Index; WC, Waist Circumference; HDL-C, high-density lipoprotein-cholesterol; LDL-C, low-density lipoprotein-cholesterol; hsCRP, high sensitive C reactive protein; HbA1c, hemoglobin A1c

### Association between risk of short telomeres and T2DM incidence

**Fig. 1:**
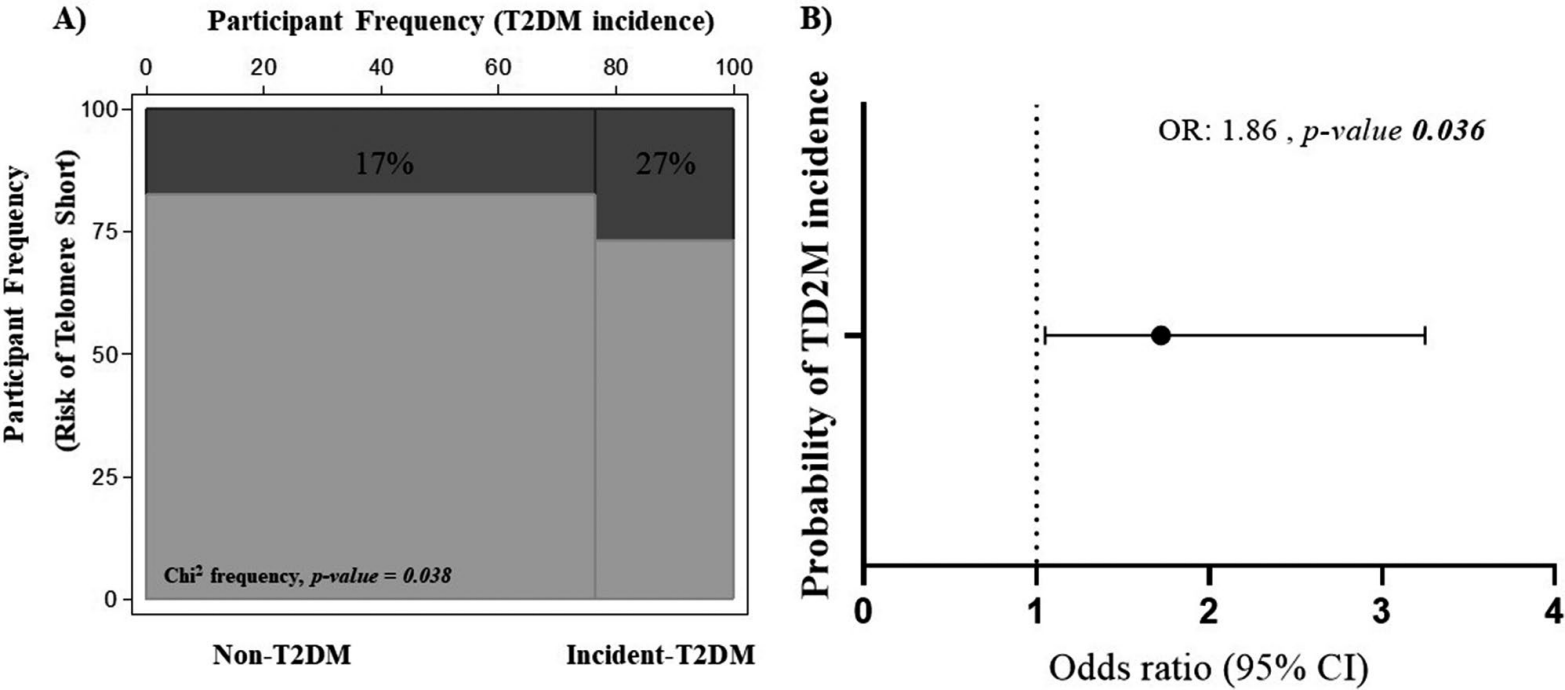
Association between risk of short telomeres and T2DM incidence. Participant frequency (**A**) and Odds Ratio (**B**) of T2DM incidence according to the risk of short telomeres. Mosaic plots: Height of bars represents proportions of participants at risk or no risk of short telomere, the width of each bar is proportional to T2DM incidence. The analysis were performed applying the Fisher’s exact test (**A**) and logistic association, Odds ratios and 95% confident intervals of T2DM incidence adjusted by age, gender BMI, WC, HbA1c, triglycerides, HDL-c and family history of both diabetes and early coronary heart disease (**B**)

We evaluated the association between short telomeres and T2DM incidence (Fig. 1) through the percentage of patients at risk of having short telomeres in a mosaic plot applying the Fisher’s exact test (Fig. 1A), and the logistic association (Fig. 1B). We found a higher percentage of patients at risk of short telomeres in the group of Incident-T2DM patients in comparison with the group of Non-T2DM patients (27% vs. 17%, respectively, *p-value* 0.038), being the odds ratio 1.86 (95% CI 1.05 to 3.25, *p-value* 0.036).

### The risk of T2DM incidence according to telomeres

**Fig. 2:**
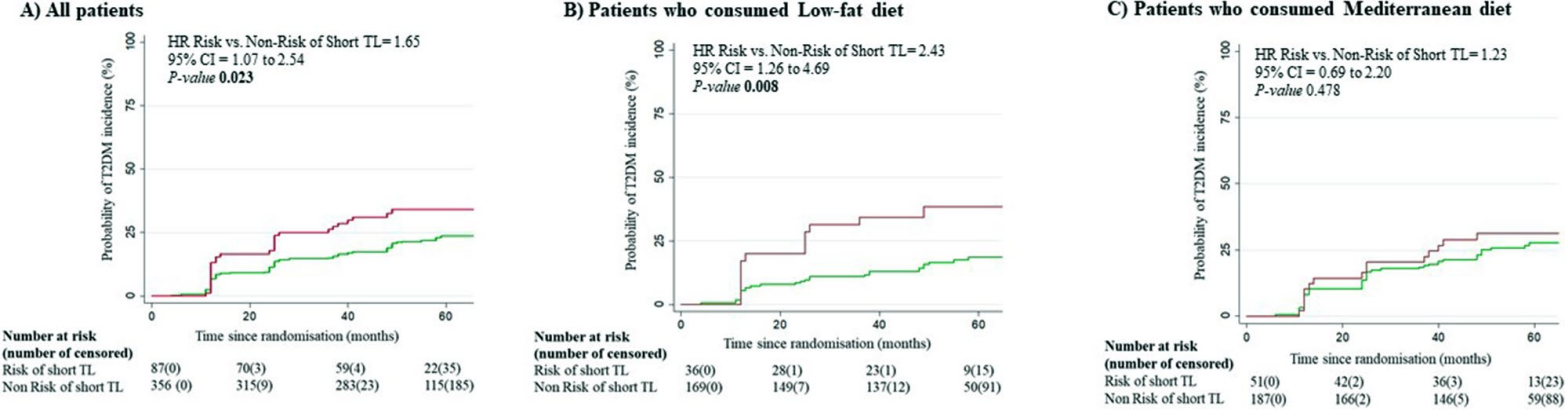
Risk of T2DM incidence by the risk of short telomeres. In the whole population (**A**) and in patients who consumed low fat diet (**B**) or Mediterranean Diet (**C**) The analysis was performed using a Cox regression curve by risk of short TL and adjusted by age, gender BMI, WC, HbA1c, triglycerides,HDL-c and family history of both diabetes and early coronary heart disease. Red line indicates risk of short telomeres and green line indicates no risk of short telomeres

The risk of T2DM incidence depending on the risk of short telomeres during dietary intervention was estimated using a Kaplan-Meier survival curve (Fig. 2). We observed that patients at risk of short telomeres had a higher incidence of T2DM than those at no risk of short telomeres, with a hazard ratio (HR) of 1.65 (95% CI 1.07 to 2.54, *p-value* 0.023, Fig. 2A). Significant interaction of diet versus risk of short telomeres was observed (*p-value* 0.048). Patients at high risk of short telomeres who consumed a low-fat diet had a higher risk of T2DM incidence compared to patients at no risk of short telomeres (HR 2.43, 95CI% 1.26 to 4.69, *p-value* 0.008) (Fig. 2B), while no differences were observed between patients at risk and those at no risk of short telomeres who consumed a Mediterranean diet (HR 1.23, 95CI% 0.69 to 2.20, *p-value* 0.478) (Fig. 2C). In addition, we performed a ROC curves analysis to evaluate the potential for TL to classify the patients in Incident-T2DM and Non-T2DM. First, based on the classic predictors of T2DM remission (i.e., BMI, age, gender, triglycerides and statins use), we observed an area under the curve (AUC) of 0.62 (95% CI, 0.56–0.69) (Additional Fig. 2A). To improve this model with our key variable, we carried out the ROC curve analysis added telomere length. Our results showed an AUC of 0.66 (95% CI, 0.60–0.72) (Additional Fig. 2B). The two models were significantly different (*p* = 0.027) through a DeLong Test.

## Discussion

Our study showed that CHD patients at risk of short telomeres had a higher risk of T2DM development than those at no risk of short telomeres. Additionally, the risk of T2DM associated with shortened telomeres was mitigated by a Mediterranean diet. Indeed, patients with risk of short telomeres at baseline showed a higher risk of developing T2DM when consuming a low-fat diet compared to patients at no risk of short telomere, while no differences were found in those patients who consumed a Mediterranean diet.

Previous research has extensively explored the link between TL and the risk, onset, and progression of T2DM. Case-control studies have shown that patients with T2DM tend to have shorter TL compared to non-diabetic subjects [25, 26]. In our study, we observed a higher percentage of patients at risk of short telomeres (< 20th percentile) among Incident-T2DM patients compared to Non-T2DM patients, with an 84% higher risk of developing T2DM in those subjects having shortened TL. Similarly, large longitudinal studies have demonstrated that healthy individuals at risk of shorter telomeres have a 30–50% independent and significantly higher risk of developing T2DM [9–11]. Taken together with our results, these findings provide support for the notion that telomere shortening may underlie mechanisms of diabetes development. Although the relationship between TL and T2DM incidence in patients with CHD has not been examined thoroughly, Piplani et al. showed the first study with a cross-sectional analysis reporting a relationship between telomere length and T2DM presence in 130 patients diagnosed with ischemic heart [27]. The coexistence of diabetes and CVD leads to a consequent increase in oxidative stress and inflammation levels, which, in turn, lead to higher mortality rates and accelerated telomere shortening [28]. In this sense, our data suggest that a process of early cellular aging, induced by oxidative stress, contributes to increasing cardiovascular complications and mortality by promoting the development of diabetes.

Because patients with CHD and T2DM have a considerably higher risk of developing a new cardiovascular event than those without T2DM [29], research into strategies, such as dietary interventions, for T2DM prevention has become an imminent requirement. Genetic differences between individuals play an important role in determining the efficacy of dietary approaches, leading to the identification of genetic markers in the context of precision nutrition. Thus, our findings propose that epigenetic markers, such as TL, could be a useful biomarker to identify patients at high risk of development common CVD comorbidities and personalize dietary strategies to decrease the risk of T2DM development.

Although several studies have evaluated the impact of lifestyle intervention on telomere shortening in the context of cancer [30], obesity [31] or CVD [23], it is currently unknown whether TL is associated to the response to lifestyle interventions. To our knowledge, this is the first study to demonstrate that the Mediterranean diet may mitigate the effects of having a short TL in CHD patients. We observed that patients at risk of short telomeres at baseline showed a higher risk of developing T2DM when consuming a low-fat diet, in contrast to those adhering to a Mediterranean diet in which no differences were found. Based on the idea that TL could reflect an individual’s cumulative exposure to inflammation and oxidative stress conditions, the Mediterranean diet, known for its rich antioxidant content, could potentially act as a protective factor against telomere shortening [32]. Cánudas et al. presented a recently systematic review and meta-analysis provides the most wide-ranging analysis to date on the positive association between Mediterranean Diet adherence and TL in blood cells. The global effect of the Mediterranean diet on TL suggested that increased adherence to a healthy patter such as Mediterranean diet can serve to counteract multiple age-related diseases by mitigating telomere shortening [33]. In the context of our study, Cano-Ibáñez et al. examined the nutritional disparities between both healthy diets (a Mediterranean and a low-fat diet), which were recommended in CORDIOPREV study. They specifically observed a lower nutritional density of mono and polyunsaturated fatty acids in the low-fat group, attributed to reduced consumption of nuts and olive oil [34]. The consumption of both fatty acids has been associated with TL in several studies, mainly [35], due to their crucial role in protecting against oxidative processes and their influence on lipid mediators that regulate inflammation, which is associated with DNA protection, including telomere integrity.

Additionally, in our study, Non-T2DM patients at risk of short TL exhibited elevated levels of HDL-c compared to Incident-T2DM patients. HDL-c exerts antioxidant and anti-inflammatory effects [36]. Reduced levels of HDL-c observed in patients following a low-fat diet (but not a Mediterranean diet), could potentially lead to greater oxidative damage in G-rich telomere repeat sequences, making these individuals more susceptible to developing T2DM.

Our results support TL being a possible biomarker with the capacity to evaluate how patients with CHD respond to specific dietary treatments in terms of T2DM prevention. Consequently, these findings contribute to reduce the probability of a new cardiovascular event. Nevertheless, our study has limitations. The primary goal of the CORDIOPREV trial was not to prevent T2DM. However, it was a secondary analysis conducted in the subgroup of CHD patients without T2DM at baseline. Therefore, it limits our findings to individuals with this comorbidity.

In conclusion, our study shows that patients with shorter TL have a higher risk of developing T2DM. This association could be mitigated with a specific dietary pattern, in our case a Mediterranean pattern, to prevent the development of T2DM in patients with CHD.

### Electronic supplementary material

Below is the link to the electronic supplementary material.

**Supplementary Material 1: Additional Table 1.** Baseline characteristics of the study population according to the incidence or non-incidence of diabetes

**Supplementary Material 2: Additional Figure 1.** Flow Chart of the Study

**Supplementary Material 3: Additional Figure 2.** T2DM incidence assessed by ROC curve models based on clinical variables and telomere length

## Data Availability

Collaborations with the CORDIOPREV Study are open to Biomedical Institutions, always after an accepted proposal for a scientific work. Depending on the nature of the collaboration, electronic data, hard copy data, or biological samples should be provided. All collaborations will be made after a collaboration agreement. Terms of the collaboration agreement will be specific for each collaboration, and the extent of the shared documentation (i.e., deidentified participant data, data dictionary, biological samples, hard copy, or other specified data sets) will be also specifically set on the light of each work.

## Abbreviations

ADA: American Diabetes Association
BMI: Body Mass Index
CI: Confident Intercal
WC: Waist Circumference
CVD: Cardiovascular Disease
CORDIOPREV study: CORonary Diet Intervention with Olive oil and cardiovascular PREVention study
CHD: Coronary Heart Disease
HbA1c: Glycosylated Haemoglobin
HR: Hazard Ratio
hs-CRP: High sensitive C-Reactive Protein
HDL-c: High-density lipoprotein-cholesterol
LDL-c: Low-density lipoprotein-cholesterol
SEM: Standard Error of the Mean
TL: Telomere Length
T2DM: Type 2 Diabetes Mellitus
