# Supplementary material for: Telomere length as biomarker of nutritional therapy for prevention of type 2 diabetes mellitus development in patients with coronary heart disease: CORDIOPREV randomised controlled trial
Source: Cardiovasc Diabetol. 2024 Mar 16;23:98. doi: 10.1186/s12933-024-02175-5 (PMC10944592; doi:10.1186/s12933-024-02175-5)
Supplement: Supplementary file 2 — Supplementary Material 2: Additional Figure 1. Flow Chart of the Study [file 12933_2024_2175_MOESM2_ESM.docx]

**Additional Figure 1. Flow Chart of the Study.**

**
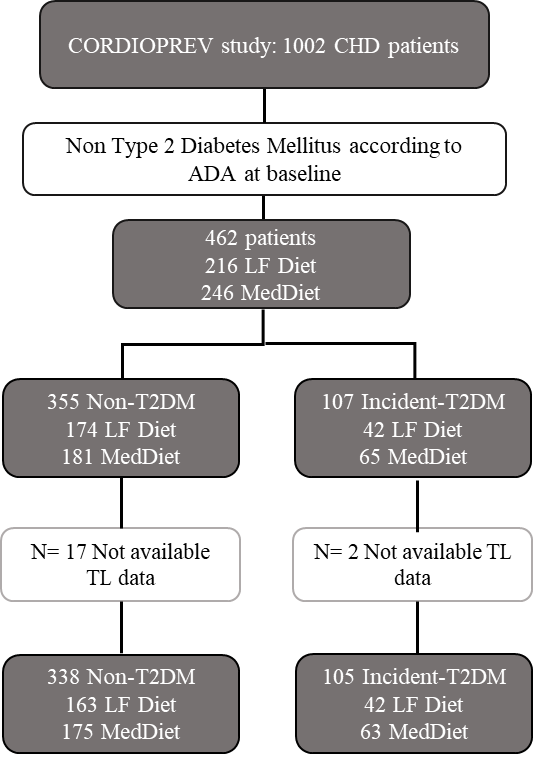
**

**Abbreviations: CHD, Coronary Heart Disease; ADA, American Diabetes Association; LF, Low-Fat; MedDiet, Mediterranean Diet; TL, Telomere Length**

**Additional Table 1. Baseline characteristics of the study population according to the incidence or non-incidence of diabetes**

|  | **Non-T2DM patients**  (n=338) | **Incident-T2DM patients**  (n=105) | ***p-value*** |
| --- | --- | --- | --- |
| *Men/Women (n)* | 288/50 | 86/19 | 0.415 |
| *LowFat/MeDiet (n)* | 163/175 | 42/63 | 0.140 |
| *Age (years)* | 57.32±0.51 | 59.00±0.87 | 0.109 |
| *Weight (kg)* | 82.32±0.71 | 85.67±1.49 | **0.028** |
| *BMI (kg/m2)* | 29.80±0.22 | 31.43±0.47 | **<0.001** |
| *WC (cm)* | 101.56±0.58 | 105.33±1.10 | **0.002** |
| *HDL-c* (mmol/L) | 44.56±0.54 | 43.46±1.05 | 0.340 |
| *LDL-c* (mmol/L) | 91.15±1.36 | 93.65±2.70 | 0.388 |
| *hsCRP* (nmol/L) | 2.49±0.19 | 2.88±0.30 | 0.322 |
| *Triglycerides* (mmol/L) | 119.26±3.29 | 131.16±6.52 | 0.088 |
| *HbA1c (%)* | 5.85±0.02 | 6.027±0.03 | **<0.001** |
| *Glucose* (mmol/L) | 92.50±0.54 | 96.17±1.05 | **0.001** |
| *Insulin* (nmol/L) | 8.35±0.32 | 10.65±0.66 | **0.001** |

Continuous variables are represented as means ± SE. Variables were compared using the analysis t-test. Abbreviations: BMI Body Mass Index; WC, Waist Circumference; HDL-C, high-density lipoprotein-cholesterol; LDL-C, low-density lipoprotein-cholesterol; hsCRP, high sensitive C reactive protein; HbA1c, hemoglobin A1c.
