# Supplementary figures and images for: Telomere length as biomarker of nutritional therapy for prevention of type 2 diabetes mellitus development in patients with coronary heart disease: CORDIOPREV randomised controlled trial
Source: Cardiovasc Diabetol. 2024 Mar 16;23:98. doi: 10.1186/s12933-024-02175-5 (PMC10944592; doi:10.1186/s12933-024-02175-5)

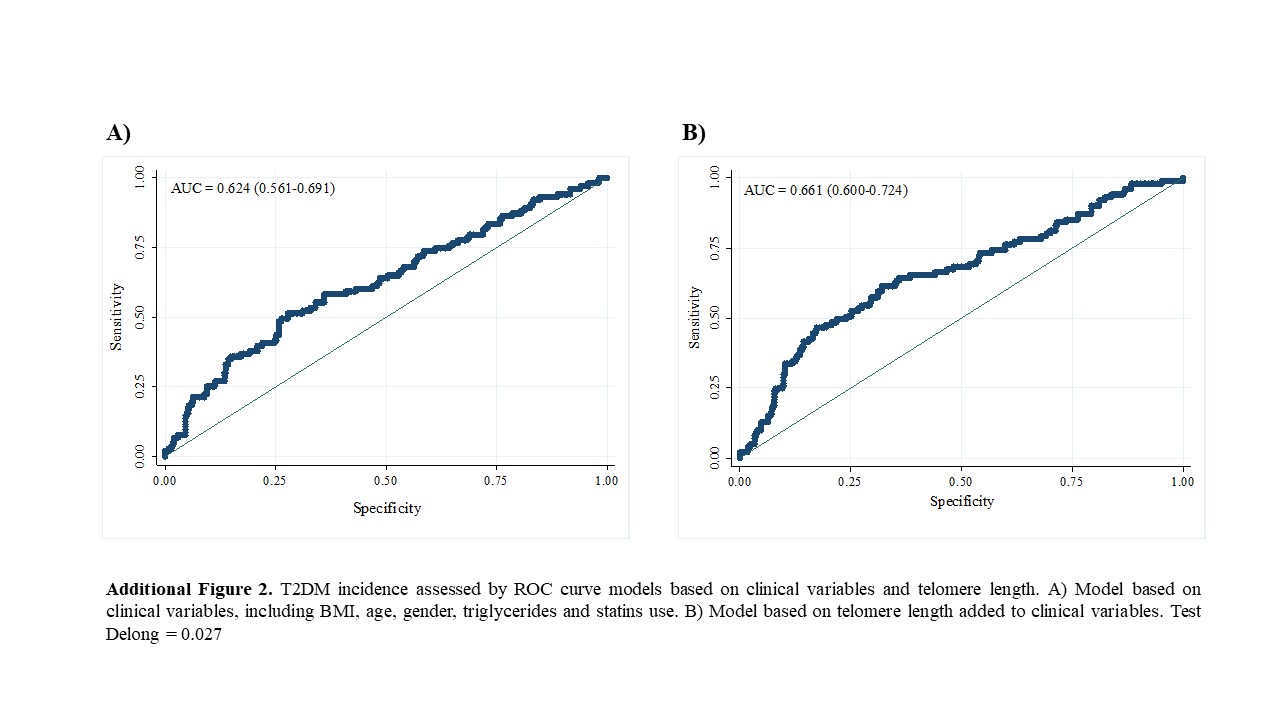

Supplement: Supplementary file 3 — Supplementary Material 3: Additional Figure 2. T2DM incidence assessed by ROC curve models based on clinical variables and telomere length [file 12933_2024_2175_MOESM3_ESM.jpg]
